# ANCA-negative microscopic polyangiitis with diffuse alveolar hemorrhage masquerading as congestive heart failure

**DOI:** 10.1186/s13317-020-00143-z

**Published:** 2021-01-06

**Authors:** Arash Mollaeian, Nina Chan, Rohit Aloor, Jeffery S. Iding, Lois J. Arend, Seyed Hootan Forghani Saeidabadi, Christopher J. Haas

**Affiliations:** 1 MedStar Health Internal Medicine Residency Program, Baltimore, MD USA; 2 Department of Pathology, MedStar Franklin Square Hospital, Baltimore, MD USA; 3 Department of Pathology, Johns Hopkins Hospital, Baltimore, MD USA; 4 Nasseri Clinics of Rheumatic and Arthritic Diseases, Baltimore, MD USA

**Keywords:** ANCA, Anti-neutrophil Cytoplasmic Antibody-Associated Vasculitis, Microscopic Polyangiitis, Vasculitis

## Abstract

**Background:**

Microscopic polyangiitis (MPA) is a subtype of anti-neutrophil cytoplasmic antibody-associated vasculitis (AAV), involving small and medium sized vessels, often affecting the kidneys and lungs. Anti-neutrophil cytoplasmic antibody (ANCA) is detected in up to 90% of cases of MPA and its detection helps guide diagnosis, however cases of ANCA-negative MPA have been reported, hence definitive diagnosis relies on tissue biopsy.

**Case report:**

A 23-year-old man was evaluated for dyspnea and pleuritic chest pain, and found to have bilateral intra-alveolar opacities and hilar adenopathy. Diagnostic work up revealed positive anti-nuclear antibodies (ANA) and negative ANCA, which in the setting of a non-classical presentation, delayed diagnosis and appropriate treatment. Due to persistent symptoms and a high suspicion for autoimmune disease with pulmonary-renal syndrome, he underwent lung biopsy which revealed intra-alveolar hemorrhage and capillaritis indicative of microscopic polyangiitis (MPA). Surprisingly, kidney biopsy was not typical of classic MPA, but revealed less common features. Due to therapeutic noncompliance he was readmitted multiple times thereafter with rare complications of MPA such as acute pancreatitis and hemorrhagic pericardial effusion with tamponade.

**Conclusion:**

This case serves as an important clinical reminder to consider AAV even in those with negative ANCA serologies and a high suspicion for pulmonary-renal syndrome. It also demonstrates the high morbidity in cases of diagnostic delay and inadequate treatment.

## Background

The 2012 Chapel Hill Consensus guideline recognizes microscopic polyangiitis (MPA) as a subset of ANCA-associated vasculitis (AAV), a group of necrotizing pauci-immune vasculitides that predominantly affects small vessels—capillaries, venules, and arterioles—with few or no immune deposits [1]. AAV commonly affects the renal and pulmonary parenchyma to varying degrees, and is characterized by the presence of antibodies against neutrophilic cytoplasmic components, Proteinase-3 (PR3, c-ANCA) and Myeloperoxidase (MPO, p-ANCA). AAV include Microscopic Polyangiitis (MPA), Granulomatosis with polyangiitis (GPA; Wegener’s) and Eosinophilic granulomatosis with angiitis (EGPA; Churg-Strauss), as well as a variety of additional AAV-associated syndromes (drug-induced vasculitis, renal-limited vasculitis (RLV)), each characterized by the presence of distinct clinical presentation, ANCA subtypes, histopathological findings, and associated laboratory findings [1–4].

MPA is characterized by the presence of anti-MPO (p-ANCA) antibodies, renal and pulmonary involvement, and the absence of granulomatous inflammation on histopathology, a defining feature when compared to GPA and EGPA which, while demonstrating both renal and pulmonary involvement, are characterized by the presence of anti-PR3 (c-ANCA) and granulomatous disease. Though a widely accepted marker for AAV, the presence of ANCA is variable and not detected in all cases. ANCA is detected in 80–90% of cases of MPA and its detection has become integral in the diagnostic workup as well as classification and prognostication of all AAV [3, 4]. While current evidence indicates that ANCA plays a role in pathogenesis of MPA and other AAV, cases of ANCA-negative vasculitis, including cases of ANCA-negative MPA, GPA, and EGPA have been reported [1–7]. We present a case of ANCA-negative MPA in a young man who presented with hemoptysis and was found to have diffuse bilateral lung infiltrates, acute kidney injury, and heart failure. Following a circuitous diagnostic course masked by symptoms of heart failure, he was eventually diagnosed with MPA, however the course of his disease was further complicated by medication non-compliance as well as rare complications of AAV, including acute pancreatitis, pancreatic pseudoaneurysm, and cardiac tamponade.

## Case report

**Fig. 1:**
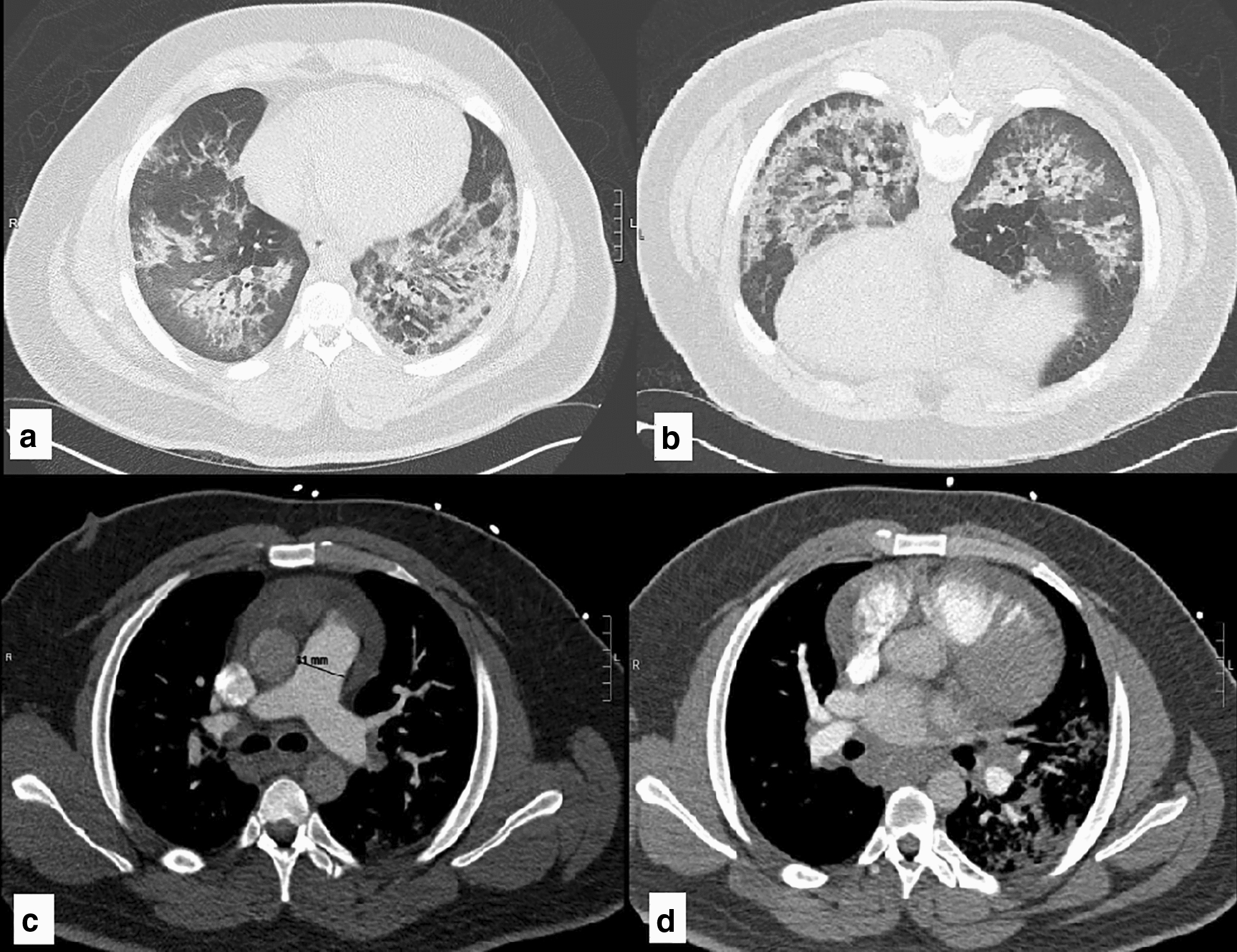
CT scans of the chest. **a** CT Scan of the chest on first day of presentation demonstrating bilateral centrilobular opacities, hilar/mediastinal lymphadenopathy, moderate cardiomegaly, a trivial pericardial effusion and enlargement of the main pulmonary artery. **b** Prone CT chest on day 7 of admission, revealing persistent intra-alveolar opacities with associated mediastinal lymphadenopathy

A 23-year-old African-American man with past medical history of G6PD-deficiency and obesity presented with dyspnea and pleuritic chest pain that improved upon leaning forward and scant hemoptysis of two weeks duration. He was notably hypertensive to the 220/110 s mmHg and tachycardic to the 120 s, but had a preserved respiratory rate and oxygen saturation. Physical exam was remarkable for bilateral diffuse wheezing throughout all lung fields. Other parts of the exam including cardiovascular, nasopharynx, skin and musculoskeletal systems were unremarkable. Additional pertinent history was remarkable only for marijuana smoking. Electrocardiogram was unremarkable except for left atrial enlargement. Chest X-ray revealed bilateral pulmonary infiltrates, cardiomegaly, and mediastinal enlargement suggestive of bulky hilar adenopathy. Computed tomography (CT) scan of the chest with contrast was performed which was negative for pulmonary embolism, but revealed bilateral centrilobular opacities, hilar/mediastinal lymphadenopathy, and a trivial pericardial effusion (Fig. 1a). Initial laboratory diagnostics were significant for acute kidney injury with a serum creatinine of 2.03 (mg/dL), trace proteinuria, troponin elevation to 0.232 (ng/mL), and a D-Dimer of 1.3 (mcg/mL). Complete blood count revealed no leukocytosis, but concomitant eosinophilia, with no evidence of anemia. The patient was subsequently admitted to the medicine wards and a therapeutic regimen of antihypertensive agents as well as intravenous ceftriaxone and azithromycin for presumed community acquired pneumonia was initiated.

Additional diagnostic evaluation demonstrated marked elevation of CRP (80 mg/L) and ESR (67 mm/hr). Pheochromocytoma screening with serum catecholamines and metanephrines was negative. An echocardiogram revealed an ejection fraction of 40%, moderate concentric left ventricular hypertrophy, and trivial pericardial effusion without evidence of tamponade or pulmonary artery hypertension. Despite continuation of antibiotics for pneumonia and adjunctive heart failure management, the patient’s clinical status failed to improve. In the setting of an unremarkable infectious workup and concern for an autoimmune etiology, the patient was started on oral prednisone 60 mg daily. The patient remained symptomatic, experiencing episodic chest pain and shortness of breath, with blood pressure lability. Repeat chest X-ray showed worsening bilateral infiltrates. Subsequently, ANA resulted weakly positive (1:160) with a homogenous pattern and rheumatoid factor (RF) returned negative.

**Fig. 2:**
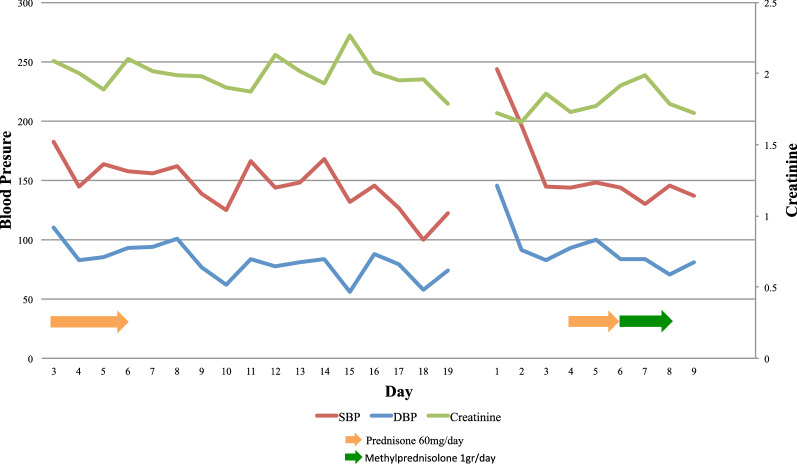
Blood pressure and creatinine during first and second hospitalizations. *SBP* Systolic Blood Pressure, *DBP* Diastolic Blood Pressure

**Fig. 3:**
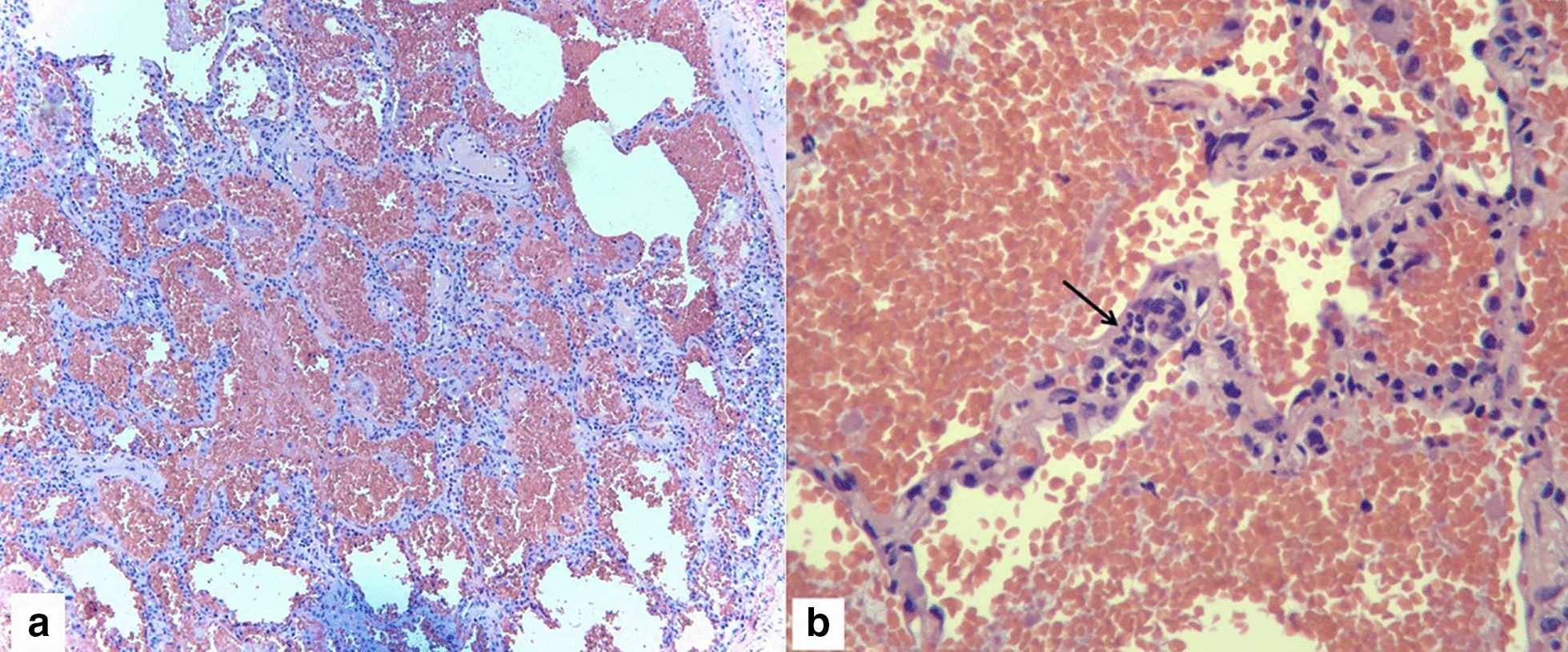
Lung Biopsy. **a** Intra-alveolar hemorrhage (100×) **b** Polymorphonuclear cells in alveolar septa; capillaritis (Arrow) (400x)

On the fifth day of admission, antibiotics were discontinued in light of negative cultures and lack of significant clinical improvement. The patient’s blood pressure was noted to be slightly improved with addition of prednisone however remained poorly controlled on multiple medications (Fig. 2). Autoimmune panel including ANCA (MPO/p-ANCA, and PR3/c-ANCA), anti-double-stranded DNA antibody, anti-Smith antibody, C3 and C4 complements, anti-histone antibody, as well as other autoimmune related factors and infectious serologies returned negative or within normal limits (Table 1). Renal ultrasound and renal artery duplex were performed which did not reveal any evidence of hydronephrosis or renal artery stenosis, respectively. A repeat high-resolution chest CT scan was obtained, both in supine and prone positions, to help differentiate early interstitial lung disease from intra-alveolar processes, which showed persistent intra-alveolar opacities without any change on prone position (Fig. 1b). Over the course of the following days, the patient reported improvement in his symptomatology and his blood pressure improved, therefore he was taken off prednisone on the sixth day. Pulmonary function testing was performed in the setting of bilateral hilar adenopathy and fixed infiltrates, which revealed a mixed obstructive and restrictive pattern. He eventually underwent lung biopsy by video-assisted thoracoscopic surgery (VATS) with right medial lobe wedge resection and was subsequently discharged with instructions to follow up with rheumatology for planned initiation of rituximab. The histopathological results of his lung biopsy demonstrated extensive intra-alveolar hemorrhage with linear polymorphonuclear (PMN) cell collections in alveolar septa and capillaritis without any evidence of granulomatous changes, indicative of microscopic polyangiitis (Fig. 3). Immunohistochemistry on lung biopsy samples was not performed and renal biopsy was deferred to outpatient rheumatology.

**Table 1:** Autoimmune and infectious workup results during first admission

Lab	Results	Lab	Results	Lab	Results
WBC	9.3	Neutrophils	75.9%	Potassium	2.9
Hgb	13.1	Lymphocytes	12.5%	Sodium	138
Hct	37.8	Eosinophils	1.3%	Creatinine	2.03
Platelet	343	Monocytes	10.1%	GFR	52
Troponin	0.232	D-Dimer	1.3	TSH	1.65
CRP	80	GBM Ab	Negative	CMV IgM	Negative
ESR	67	SS-A/Ro	Negative	Cox A & B Ab	Negative
ANA	1:160 (Homogenous)	SS-B/La	Negative	Cryptococcal Ag	Negative
RF	< 10	ACA (IgG, IgM)	< 9	EBV DNA	Negative
Anti-CCP	4	TTG (IgA, IgG)	1, 3	HBV DNA	Negative
ANCA	Negative	Ribosome IgG	3	HCV RNA	Negative
Anti ds-DNA	Negative	Endomysial IgG	< 1:10	HIV 1&2 Ab	Negative
Anti-Smith Ab	Negative	Anti Gliadin	< 7	Parvovirus B19	Negative
Histone auto Ab	Negative	Anti Gliadin	< 2	Syphilis (RPR/FTA)	Negative
Scl-70 Ab	Negative	Free Kappa	1.73	HHV6 DNA	Negative
C3	130	Free Lambda	1.49	Lyme ELISA	Negative
C4	32.4	K/L Ratio	1.16	Histoplasma Ag	Negative
IgA	250	IgG	1160	IgM	1110

*WBC* White Blood Cell, *Hgb* Hemoglobin, *Hct* Hematocrit, *GFR* Glomerular Filtration Rate, *TSH* Thyroid Stimulating Hormone, *Ab* Antibody, *Ig* Immunoglobulin, *ESR* Erythrocyte Sedimentation Rate, *CRP* C-Reactive Protein, *ANA* Anti-Neutrophil Antibody, *ANCA* Anti-Neutrophil Cytoplasmic Antibody, *Scl* Scleroderma, *C3* Complement 3, *C4* Complement 4, *ds-DNA* Double-Stranded DNA, *RF* Rheumatoid Factor, *CCP* Cyclic Citrullinated Peptide, *SS-A* Sjӧgren-Syndrome-related-antigen A, *SS-B* Sjӧgren-syndrome-related-antigen B, *ACA* Anti Cardiolipin Ab, *TTG* Tissue trans-glutaminase, *HIV* Human Immunodeficiency Virus, *HCV* Hepatitis C Virus, *HBV* Hepatitis B Virus, *CMV* Cytomegalovirus, *EBV* Epstein-Barr Virus, *Cox A & B* Coxsackievirus A & B, *HHV* Human Herpes Virus, RPR: Rapid Plasma Reagin, *FTA* Fluorescent Treponemal Antibody

**Fig. 4:**
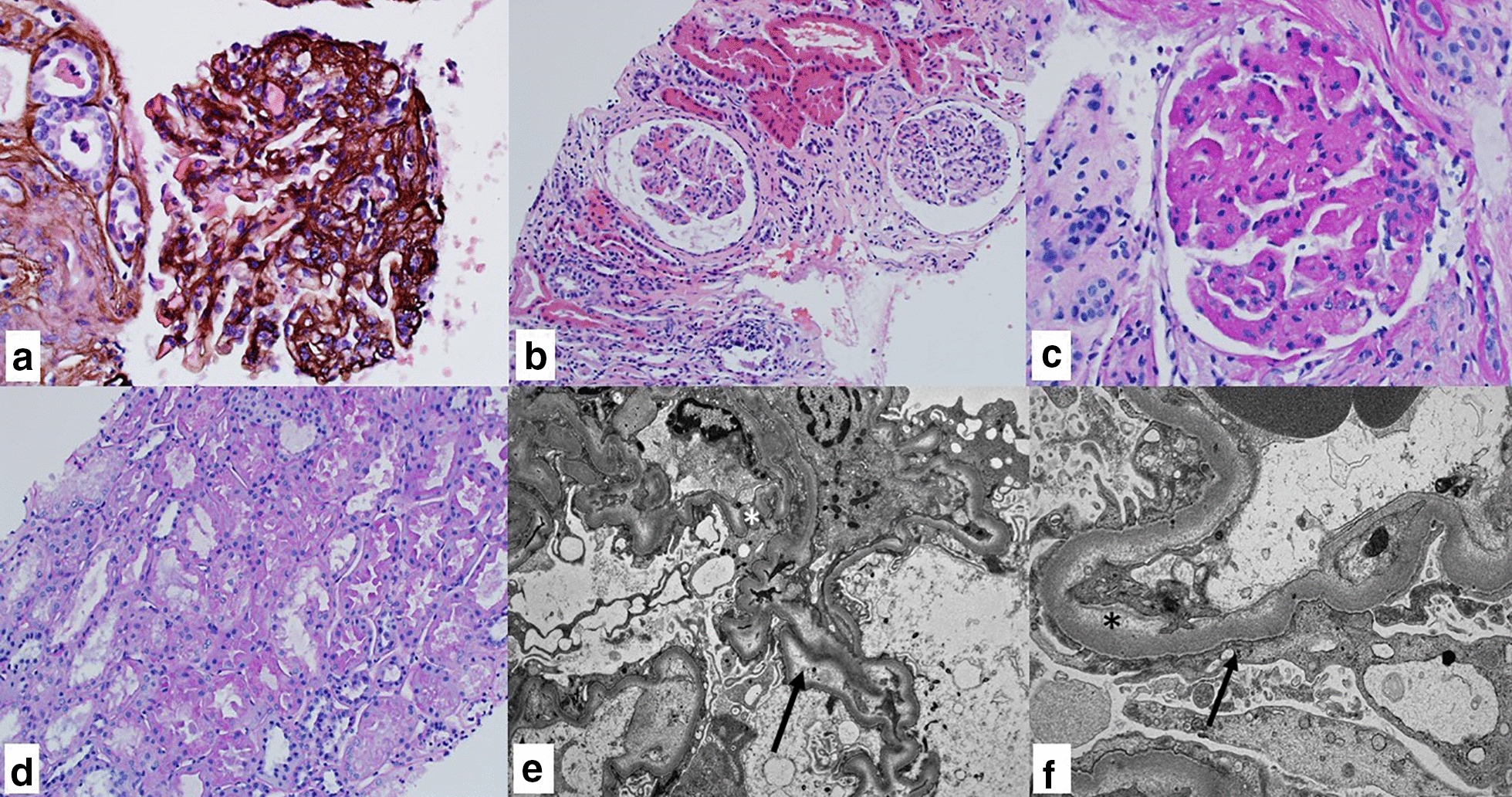
Kidney Biopsy. **a** Glomerulus with segmental sclerosis of the right half of the tuft, Periodic Acid-Methenamine Silver (PASM, 200×) **b** One glomerulus (left) with an ischemic appearance including capillary wall wrinkling and tuft retraction, other glomerulus (right) has mild hypercellularity including a few neutrophils (H&E, 100×) **c** A glomerulus with a bloodless appearance, obliteration of capillary lumens and thickening of capillary walls with segmental duplication (Periodic Acid-Schiff (PAS), 200×) **d** Tubules show cytoplasmic vacuolation, apical blebbing, thinning, and focal simplification. The interstitium contains a sparse lymphocytic infiltrate (PAS, 100×) **e** Electron micrograph of glomerular tuft showing ischemic-type capillary wall wrinkling (asterisk) and diffuse subendothelial electron lucent widening (arrow) **f** Electron micrograph showing podocyte foot process effacement (arrow) and mild subendothelial electron-lucent widening (asterisk)

Unfortunately, the patient was lost to follow up, failing to attend any of the scheduled appointments. He was subsequently readmitted six months later with similar symptoms, and once again found to be in hypertensive emergency, complicated by acute decompensated heart failure. Repeat echocardiogram revealed a further decline in his ejection fraction to 25% and a trivial pericardial effusion. Rheumatology was consulted and recommended pulse steroid therapy with methylprednisolone 250 mg four times a day for three days, which led to significant improvement in symptoms and blood pressure. Repeat ANCA and autoimmune serological workup remained negative. Following initiation of pulse steroid therapy, he underwent renal biopsy. The biopsy contained forty glomeruli, six of which were globally sclerotic. One glomerulus had segmental sclerosis. There were changes suggestive of microangiopathic injury in some arteries and glomeruli, such as bloodless glomeruli and mild intimal myxoid changes in arteries, with associated acute tubular injury. Focal tubular atrophy and interstitial fibrosis were estimated to involve approximately 10% of the cortex. No evidence of crescents or granulomatous changes were observed. Immunofluorescence was unremarkable, with only non-specific linear glomerular and tubular basement membrane staining for IgG, kappa, lambda, and albumin, indicative of pauci-immunity. Electron microscopy demonstrated segmental effacement of podocyte foot processes (40%) with vacuolation and microvillous transformation of the podocyte cytoplasm, ischemic-type capillary wall wrinkling, and subendothelial electron lucent widening (Fig. 4). He was subsequently discharged on a steroid taper with plans for outpatient rituximab. Despite extensive education about his condition he was once again lost to follow up and missed appropriate treatment.

**Fig. 5:**
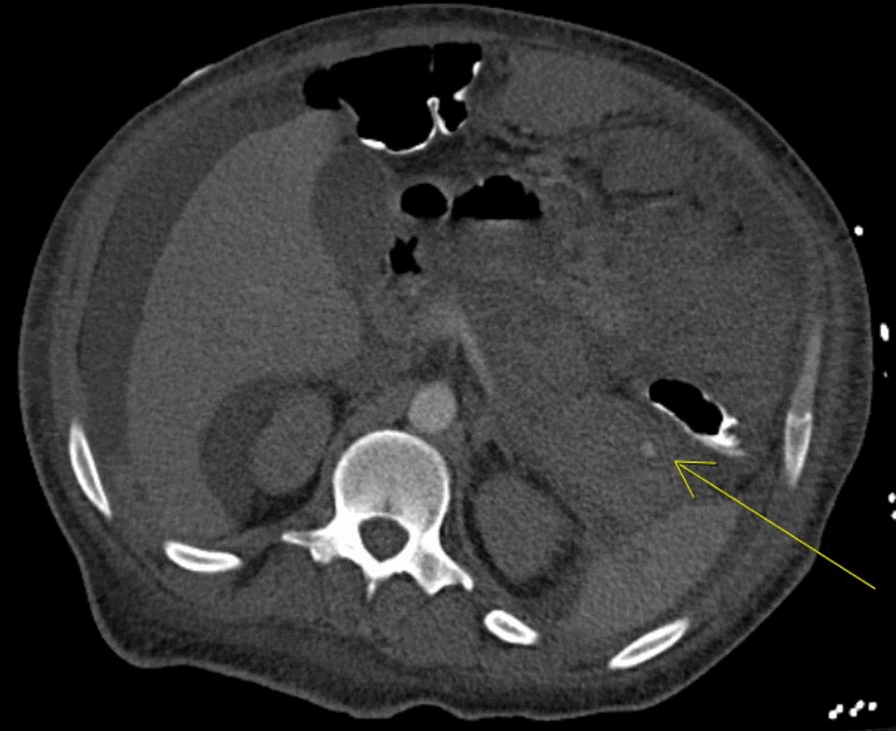
CT angiography of the Abdomen. A 7 mm arterially enhancing focus in the tail of the pancreas that follows the blood flow (Arrow), consistent with a pseudoaneurysm, with an associated large left upper quadrant hematoma and small amount of hemorrhage in the right paracolic gutter

**Fig. 6:**
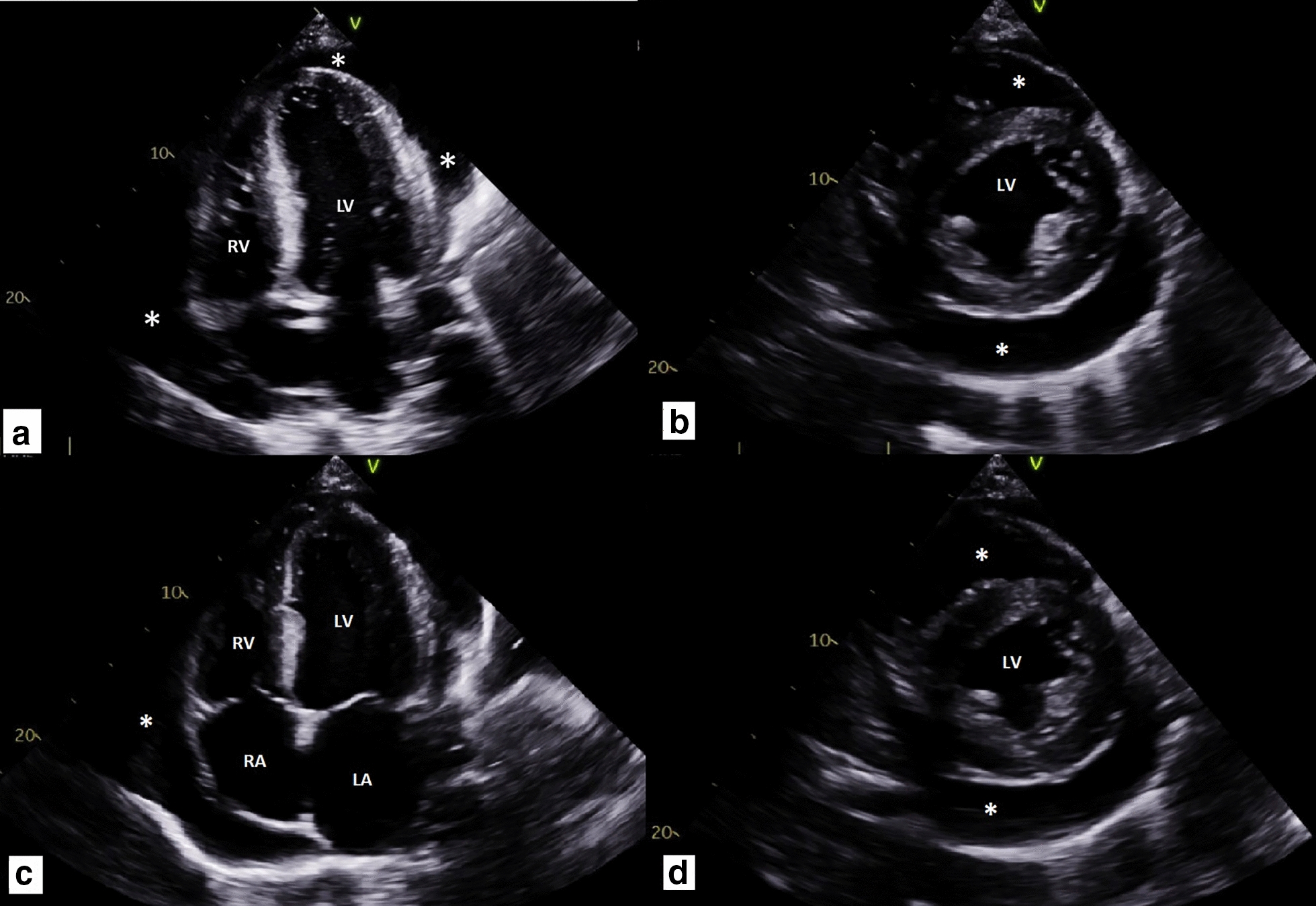
Echocardiogram. Four chamber views in diastole **a** and systole (**c**). Short axis views in Diastole **b** and systole (**d**). Echocardiography demonstrating a large circumferential pericardial effusion (Asterisks) without hemodynamic compromise, mildly dilated left ventricle (LV) with normal wall thickness, and severe globally depressed systolic function with left ventricular ejection fraction of 10%, normal right ventricle (RV) size and mildly reduced right ventricular systolic function

Over the course of the next year, he was readmitted on multiple occasions and eventually progressed to dialysis-dependent end-stage renal disease. His two most recent admissions were characterized by pancreatitis with pancreatic pseudoaneurysm complicated by retroperitoneal hematoma and cardiac tamponade, respectively. With respect to his pancreatitis, he presented in the context of acute onset abdominal pain with associated nausea and vomiting. Laboratory diagnostics demonstrated an elevated lipase (3400), normal triglycerides, and a negative IgG4 serology. Abdominal CT was significant for peripancreatic fat stranding and inflammatory changes without evidence of gallstones or biliary ductal dilation. He denied recent alcohol use. Pancreatitis course was further complicated by retroperitoneal hematoma, which was found to be secondary to a 7 mm pancreatic pseudoaneurysm rupture, as revealed on repeat abdominal CT angiography (Fig. 5). He was managed conservatively and subsequently discharged. One month later he presented with dyspnea, chest pain and nausea, and was found to have large pericardial effusion with an acute decrease in ejection fraction to 10% (Fig. 6). He underwent pericardiocentesis with removal of one liter of serosanguineous fluid. Fluid analysis was indicative of hemorrhagic etiology and negative for malignancy. On review of most recent outpatient records, the patient has been maintained on high-dose suppressive steroid therapy with plans for initiation of Rituximab or Cyclophosphamide.

## Discussion

Since the sentinel publications on ANCA in early 1980s and the landmark article by van der Woude et al. [5], our understanding of ANCA has significantly expanded. ANCA is currently recognized as having a central role in the pathogenesis of necrotizing vasculitis and glomerulonephritis particularly AAV, however the exact mechanism, and if a direct pathogenic role exists for ANCA, remains to be elucidated [8]. Recently an association between ANCA-induced neutrophil activation, regulated necrosis (necroptosis), neutrophil endothelial trap (NET) generation, complement activation, and endothelial cell damage leading to vasculitis and necrotizing glomerulonephritis has been identified in AAV by Schreiber et al. [9]. Nonetheless, detection of ANCA has become an essential part of the routine workup for vasculitis and many other autoimmune disorders. Currently only two ANCA antigens, PR3/ c-ANCA and MPO/ p-ANCA, critical components for neutrophil-mediated innate immunity, have been recognized to be clinically significant for AAV amongst a diverse array of ANCA antigens [9]. While indirect immunofluorescence testing on ethanol-fixed neutrophils is the reference method for ANCA detection and screening, positive results prompt specific testing for PR3 and MPO. As new ANCA detection methods have developed, the most recent revised international consensus recommends high-quality antigen-specific assays for PR3-ANCA and MPO-ANCA should be used as the primary screening method for ANCA, and if results for both are negative with strong clinical suspicion for small-vessel vasculitis, other immunoassays, indirect immunofluorescence, or referral to an experienced lab is recommended [10].

ANCA is detected in 80–90% of cases of MPA out of which 70% are positive for MPO-ANCA [3–6, 11–14]. Seronegative ANCA, also known as pauci-immune vasculitis, is similar to seronegative lupus or seronegative rheumatoid arthritis and the absence of specific markers may lead not only to a delay in diagnosis, but also misdiagnosis [1, 2]. Furthermore, ANCA, though initially negative, may become positive later in the course of disease or may not be detectable with currently available methods [3–5]. On the other hand, antibodies to antigens other than PR3 and MPO have been detected and are referred to as minor ANCA antigens [15]. Interestingly these antibodies are frequently detected in sera of MPO- and PR3-ANCA negative patients, and while they can mimic c-ANCA or p-ANCA patterns, they are detected in other types of small vessel vasculitis such as cocaine-induced vasculitis mimicking GPA as well as other autoimmune diseases such as Sjӧgren, rheumatoid arthritis, and ulcerative colitis [9, 15]. It is noteworthy to mention that ethnic differences between MPO and PR3 ANCA subtypes have also been observed with PR3 predominance in Japanese and Chinese ethnicity and MPO predominance in Northern Europeans and Middle-Eastern/Turkish groups [7].

Classically MPA presents with acute onset of rapidly progressive glomerulonephritis, however presentation is often atypical. Most commonly, patients present with constitutional symptoms—fever, weight loss, fatigue—over an acute or chronic course over weeks to months [3–5]. The most common age of presentation is in 5–6th decades of life with a slight male predominance [3, 4]. Multiple organs can be involved with the most common organs being kidneys and lungs, followed by skin, gastrointestinal, cardiac and nervous systems [5, 6, 11]. Renal involvement is reported in 80–100% of MPA cases and is classically characterized by rapidly progressive glomerulonephritis (GN), however renal involvement on initial presentation may range from minimal proteinuria to end-stage renal disease requiring dialysis [5]. Pauci-immune necrotizing and crescentic GN is the classic finding on biopsy in systemic AAV [12]. Crescentic involvement of the glomeruli is the most common form of glomerular involvement in MPA and is visualized in up to 90% of cases, however interstitial nephritis and tubular atrophy, as observed in this case, are reported on biopsies as well [1–5]. Berden et al. proposed a classification criteria for AAV GN, based on the pathologic features of the glomerulus, into four major groups of focal, crescentic, mixed and sclerotic [12]. Considering the Berden’s classification, our patient would fall into the focal subgroup with more than 50% normal glomeruli, which have a relatively preserved kidney function and an overall more favorable outcome compared to other pathologic subgroups [12]. In another study of ANCA-negative patients with pauci-immune renal vasculitis, 29% of renal biopsies revealed normal glomeruli and only 50% had crescents on histology [14].

Pulmonary involvement can occur in up to 55% of cases of MPA and mostly present as dyspnea, cough, and hemoptysis. Classically MPA is associated with diffuse alveolar hemorrhage and a pauci-immune, hemorrhagic necrotizing alveolar capillaritis characterized by an absence of granulomatous changes on lung and renal biopsy [5, 6], similar to what was observed in our patient (Fig. 3). Diffuse alveolar hemorrhage, caused by pulmonary capillaritis, is considered a serious complication and if accompanied by glomerulonephritis (pulmonary-renal syndrome) portends a poor prognosis [5, 6]. Pulmonary function testing in patients with MPA can show either obstructive or restrictive pattern with reduced carbon monoxide diffusion capacity (DLCO) [5].

Gastrointestinal bleeding has been reported in up to 29% of MPA patients [5], however additional manifestations such as colonic ulceration, bowel ischemia, perforation, and arterial aneurysms have also been reported [5]. Pancreatic involvement in MPA is rare and may present as acute pancreatitis or pancreatic mass [16]. In a study of 62 patients with small-medium vessel vasculitis, Pagnoux et al. demonstrated that only 3 patients had pancreatitis as a complication, with only one having MPA [17]. Cardiac involvement in patients with MPA is reported to occur in 17–50% of cases with pathophysiology similar to that of other organ systems such as lung and kidney [18, 19]. Cardiac involvement may present as hypertension, heart failure, pericarditis, and less commonly myocardial infarction [10, 18].

Management of MPA and other AAV’s includes an induction phase followed by a maintenance phase. Classic induction strategies include oral corticosteroids (1 mg/kg daily) and cyclophosphamide (2 mg/kg daily). Rituximab and cyclophosphamide are considered the drug of choice for both induction and maintenance of remission. Rituximab has been shown to be non-inferior to cyclophosphamide by the RAVE and RITUXVAS trials [3–6, 11–13]. The NORAM study revealed methotrexate was as effective as cyclophosphamide with regards to induction of remission in mild disease however relapse rates were higher for methotrexate [3–5]. Plasmapheresis has been used in cases of aggressive AAV and was shown to have better renal outcomes in MEPEX trial, however PEXIVAS trial (Plasma exchange and glucocorticoids for treatment of AAV), the largest trial in AAV so far, failed to show a significant difference in all-cause mortality or end-stage renal disease, and plasmapheresis use remains controversial [3, 4, 20]. Maintenance therapy is generally achieved by less toxic agents such as rituximab, azathioprine, and methotrexate, with rituximab becoming more and more favored in recent years. Other immunosuppressive agents like leflunomide, mycophenolate mofetil, and trimethoprim-sulfamethoxazole have been used and studied as maintenance therapy strategies as well [3–6, 11].

This case was differentiated from other potential autoimmune disorders such as systemic lupus erythematosus, Sjӧgren syndrome, sarcoidosis, and other small to medium sized vessel vasculitis such as granulomatosis with polyangiitis, polyarteritis nodosa, IgA vasculitis and cryoglobulinemic vasculitis based on presentation, laboratory and imaging findings and biopsy of the lung revealing capillaritis without granulomatous changes. Absence of pathognomic noncaseating granulomas on lung biopsy, ruled out sarcoidosis. In the light of absence of certain characteristics such as palpable purpura, as well as absence of immune complexes on tissue biopsy, immune complex vasculitides such as anti-glomerular basement membrane disease, IgA and cryoglobulinemic vasculitis were ruled out. While differentiation of GPA from MPA is difficult and they tend to share characteristics of AAV disease spectrum, the absence of granulomatous lesions on both biopsies and lack of certain classical features of GPA made it less likely in this case. EGPA was also deemed less likely considering lack of peripheral eosinophilia and tissue eosinophilic infiltration. We also believe the kidney biopsy results were affected by pulse steroids and possible steroid consumption in between admissions, however pauci-immunity and absence of granulomatous changes, and immune complex deposition favors MPA over other vasculitides.

## Conclusion

MPA is differentiated not only by the presence of anti-MPO (p-ANCA) but also histopathologically by non-granulomatous lesions and capillaritis. ANCA serologies, however, may be negative in AAV specifically MPA in as many as 10-20% of cases. This case underscores the importance of identifying pulmonary-renal syndrome and its association with AAV, the need to maintain a high index of suspicion in cases of negative ANCA serologies, and to pursue tissue biopsy despite negative serologies in such circumstances. This case further serves to demonstrate the rapid clinical deterioration and high morbidity in those cases of AAV with delayed diagnosis or inadequate treatment.

## Abbreviations

MPA: Microscopic Polyangiitis
ANCA: Anti-neutrophil cytoplasmic antibody
AAV: ANCA-associated Vasculitis
GPA: Granulomatosis with polyangiitis
EGPA: Eosinophilic granulomatosis with angiitis
G6PD: Glucose 6 Phosphate Dehydrogenase
CT: Computed Tomography
IV: Intravenous
CRP: C-reactive Protein
ESR: Erythrocyte sedimentation Rate
CAP: Community Acquired Pneumonia
ANA: Anti-Neutrophil Antibody
RF: Rheumatoid Factor
FVC: Forced Vital Capacity
FEV: Forced Expiratory Volume
VC: Vital Capacity
FRC: Forced Residual Capacity
RV: Residual Capacity
TLC: Total Lung Capacity
DLCO: Diffusing Capacity of Lung for Carbon Monoxide
VATS: Video-Assisted Thoracoscopic Surgery
PMN: Polymorphonuclear
FSGS: Focal Segmental Glomerulonephritis
IgG: Immunoglobulin G
NET: Neutrophil Endothelial Trap
